# A Gestational High Protein Diet Affects the Abundance of Muscle Transcripts Related to Cell Cycle Regulation throughout Development in Porcine Progeny

**DOI:** 10.1371/journal.pone.0034519

**Published:** 2012-04-09

**Authors:** Michael Oster, Eduard Murani, Cornelia C. Metges, Siriluck Ponsuksili, Klaus Wimmers

**Affiliations:** 1 Research Unit Molecular Biology, Leibniz Institute for Farm Animal Biology (FBN), Dummerstorf, Germany; 2 Research Unit Nutritional Physiology, Leibniz Institute for Farm Animal Biology (FBN), Dummerstorf, Germany; 3 Research Group Functional Genomics, Leibniz Institute for Farm Animal Biology (FBN), Dummerstorf, Germany; National Cancer Institute, United States of America

## Abstract

**Background:**

In various animal models pregnancy diets have been shown to affect offspring phenotype. Indeed, the underlying programming of development is associated with modulations in birth weight, body composition, and continual diet-dependent modifications of offspring metabolism until adulthood, producing the hypothesis that the offspring's transcriptome is permanently altered depending on maternal diet.

**Methodology/Principal Findings:**

To assess alterations of the offspring's transcriptome due to gestational protein supply, German Landrace sows were fed isoenergetic diets containing protein levels of either 30% (high protein - HP) or 12% (adequate protein - AP) throughout their pregnancy. Offspring muscle tissue (*M. longissimus dorsi*) was collected at 94 days post conception (dpc), and 1, 28, and 188 days post natum (dpn) for use with Affymetrix GeneChip Porcine Genome Arrays and subsequent statistical and Ingenuity pathway analyses. Numerous transcripts were found to have altered abundance at 94 dpc and 1 dpn; at 28 dpn no transcripts were altered, and at 188 dpn only a few transcripts showed a different abundance between diet groups. However, when assessing transcriptional changes across developmental time points, marked differences were obvious among the dietary groups. Depending on the gestational dietary exposure, short- and long-term effects were observed for mRNA expression of genes related to cell cycle regulation, energy metabolism, growth factor signaling pathways, and nucleic acid metabolism. In particular, the abundance of transcripts related to cell cycle remained divergent among the groups during development.

**Conclusion:**

Expression analysis indicates that maternal protein supply induced programming of the offspring's genome; early postnatal compensation of the slight growth retardation obvious at birth in HP piglets resulted, as did a permanently different developmental alteration and responsiveness to the common environment of the transcriptome. The transcriptome modulations are interpreted as the molecular equivalent of developmental plasticity of the offspring that necessitates adaptation and maintenance of the organismal phenotype.

## Introduction

Intrauterine growth retardation (IUGR), i.e., impaired prenatal growth and development, can be caused by an adverse nutritional intrauterine environment characterized by limited or excess protein supply during pregnancy [1], [2]. IUGR is a major concern in animal breeding, because of its negative impact on production [3]. Low birth weight piglets grow slower and are prone to impaired carcass and meat properties, including higher drip losses, higher body fat contents, lower muscle mass, lower glycogen reserves and lower tenderness scores when compared with their high birth weight littermates [4]–[6]. Muscle tissue of low birth weight piglets is hypothesized to develop increased hypertrophy due to reduced myofiber proliferation, which might affect fat deposition and energy metabolism [5]. Therefore, cellular growth and regulation of the cell cycle may be central in the underlying molecular mechanism governing the intrauterine adaptive response to adverse environmental conditions (“fetal programming”).

Fetal programming has recently come under heavy investigation. Evidence indicates that diet-dependent permanent consequences on the phenotype involve sophisticated modulations of the gene expression machinery, leading to a diet-specific tuning of the transcriptome in rodents [7], [8], pigs [9] and cattle [10]. Notably, it was shown that long-term modulations of gene expression will also take place due to adverse feeding regimes at crucial prenatal time periods, e.g. around conception [11] and early gestation [12]. However, modulation of the transcriptome occurs in response to exogenous effects; such changes may induce and represent adaptive processes to maintain homoeostasis and force the expression of the organismal phenotype within physiological norms. In fact, in our previous work, the progeny of sows that received either high protein levels or adequate protein levels during pregnancy only showed slight divergences in birth weight and body composition at birth [2], [13]. At later stages offspring of both groups became similar in terms of organismal phenotype, including body weight, body composition, and cellularity of muscle and adipose tissue [13], [14]. Monitoring the transcriptome at different developmental stages in the context of fetal programming, i.e., variable conditions at prenatal time points, can uncover mechanisms behind prenatal events that affect postnatal development.

We have previously shown that, depending on the gestational diet, the expression profile of a central metabolic organ like liver is affected at both prenatal and postnatal stages, specifically as an altered responsiveness of energy and nutrient-sensing pathways [9]. Here, we focus on muscle tissue; muscle represents the largest peripheral consumer of and storage hub for energy and nutrients, contributes to the species-typical shape of the body, and is a main agricultural product for human consumption when muscle becomes meat. We aimed to identify molecular pathways with relevance to the fetal initiation of postnatal growth and development in pigs. Pregnant German Landrace gilts were fed isoenergetic diets containing either adequate or excess levels of protein at the expense of carbohydrate supply. Gene expression profiles of their fetuses and offspring were investigated in a longitudinal experimental design. Transcriptome analyses indicate that maternal protein supply and/or shortage of exogenous carbohydrates triggers a programming of the offspring's genome characterized by the adjustment of molecular routes related to cell cycle regulation and proliferation. These modulations contribute to the maintenance of the overall organismal phenotype of the offspring independent of prenatal nutritional experiences.

## Results

**Figure 1:**
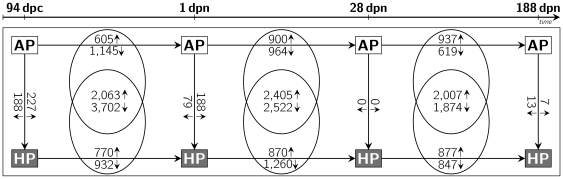
Number of probe sets showing a significantly altered abundance in muscle tissue. The number of altered probe sets between adjacent developmental stages in AP or HP offspring are indicated at horizontal arrows; the number of commonly altered probe sets between stages in AP and HP offspring are indicated at intersections; the number of probe sets showing a different abundance between HP and AP offspring at the same developmental stage are indicated at vertical arrows; small arrows at the numbers indicate a higher or lower probe set abundance, respectively.

**Figure 2:**
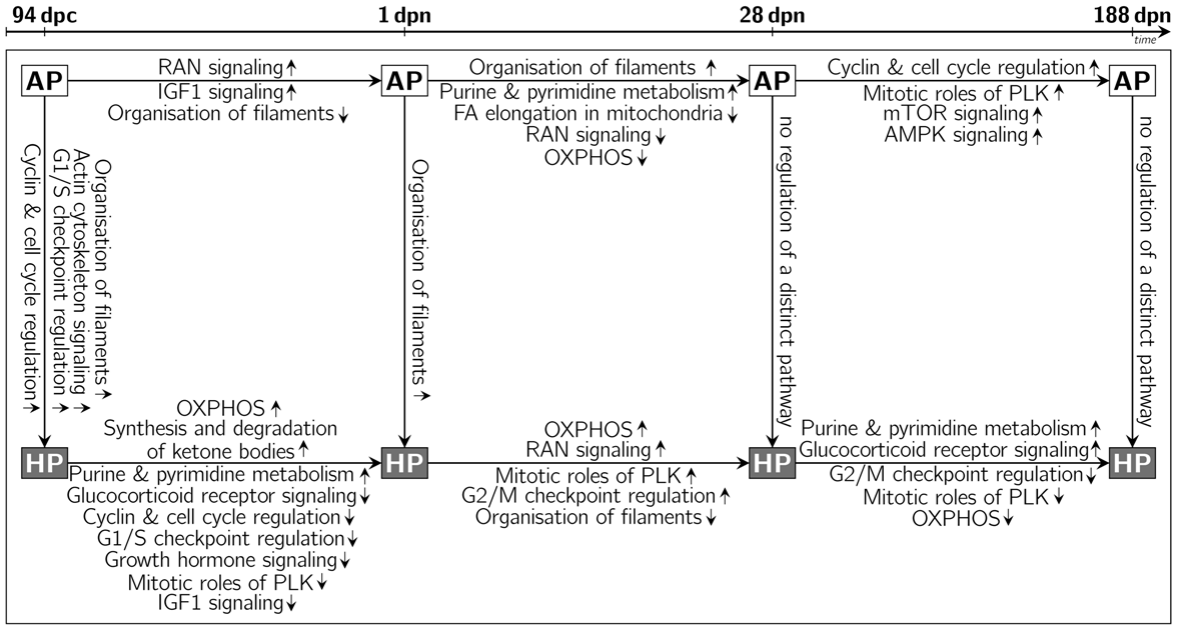
Affected pathways in muscle tissue between developmental stages and diets. Listed pathways between AP stages (white boxes) indicate shifts during development that are not found in HP offspring (black boxes) at the corresponding period. Pathways between HP stages indicate alterations that occur in HP offspring but not in AP offspring in the corresponding period. (Arrows between boxes show direction of comparison; small arrows indicate higher and lower transcript abundance, respectively. OXPHOS, oxidative phosphorylation; PLK, Polo-like kinase; mTOR, mammalian target of rapamycin; AMPK, AMP-activated protein kinase; IGF1, insulin-like growth factor 1; FA, Fatty acid; RAN, Ras-related nuclear protein).

To develop an index of genes responsive to gestational diets and to characterize molecular pathways and mechanisms related to fetal programming, we analysed diet-dependent expression patterns in a longitudinal holistic study of muscle tissue collected from progeny of sows fed either isoenergetic high-protein (HP; 30% crude protein; protein: carbohydrate ratio 1∶1.3) or adequate-protein diets (AP; 12% crude protein; protein: carbohydrate ratio 1∶5). Offspring muscle gene expression was determined at one prenatal and three postnatal stages using porcine 24 k microarrays. Transcriptional alterations of genes and resulting metabolic pathways were identified according to the variance component diet
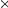
stage. Therefore, transcriptional shifts between HP and AP animals at each developmental stage (Figures 1 and 2, vertical arrows) as well as transcriptional shifts between adjacent stages among the experimental groups (Figures 1 and 2, horizontal arrows) were considered.

In comparing HP vs. AP, 15,738 probe sets overall were found to be expressed in muscle tissue according to MAS5 analysis. Further filtering based on the variability of expression of probe sets revealed 14,267 probe sets for further analysis. These probe sets represent 9,666 genes according to the recent annotation [15].

### Comparisons between HP and AP within stages

Expression profiles in HP and AP offspring were compared within each developmental stage. At 94 dpc, 465 probe sets were significantly different between HP and AP fetuses (227 HP
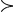
AP; Figure 1). Ingenuity Pathway Analysis indicated enrichment of molecular routes related to cyclin and cell cycle regulation, G1/S checkpoint regulation, and actin cytoskeleton signaling and organization of filaments, which were found to be more abundant in HP offspring (Table 1, Figure 2). In perinatal piglets (1 dpn), 267 probe sets differed between HP and AP offspring (188 HP
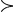
AP). The abundance of mRNA of genes associated with organisation of filaments was found to be increased in HP offspring. Remarkably, in infant piglets at weaning (28 dpn), no probe sets differed between HP and AP offspring. In young adult pigs (188 dpn) 20 probe sets were significantly different between HP and AP offspring (7 HP
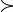
AP). Notably, probe sets representing two genes (SMAD4, CDC27) related to canonical pathways of G1/S checkpoint regulation and mitotic roles of Polo-like kinase were less abundant in HP offspring at 188 dpn.

**Table 1:** Functional annotation of muscle transcripts showing altered abundance depending on the dietary group (HP vs. AP) within different developmental stages (Ingenuity Pathway Analysis).

Developmental stage	Affected pathway	Expression	*P* value	No. of genes involved	Genes involved in pathway
94 dpc	Actin cytoskeleton signaling	up	1.02*E-2	7	ACTA1, CDC42, MSN, RDX, SSH2, TTN, VCL
	G1/S checkpoint regulation	up	2.48*E-2	3	CDK6, E2F2, HDAC6
	Cyclin and cell cycle regulation	up	1.19*E-2	4	CDK6, E2F2, HDAC6, PPP2R1A
	Organisation of filaments	up	1.53*E-4	8	COL1A1, COL1A2, COL5A1, LOX, MSN, NCK2, RDX, RHOB
1 dpn	Organisation of filaments	up	1.03*E-7	10	AKAP2, COL1A1, COL1A2, COL5A1, COL5A2, DCN, FN1, LOX, P4HA1, SERPINH1
28 dpn	-	-	-	-	-
188 dpn	-	-	-	-	-

Up and down indicate higher and lower abundance in HP compared to AP, respectively. P-value: significance of association between dataset and IP-pathways; Fischer's exact test.

### Differences of longitudinal developmental changes of transcript abundance among HP and AP offspring

Expression patterns of two adjacent developmental stages within each dietary group were compared to determine transcripts showing an altered abundance between stages (P
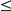
0.05) (Figure 1). Resulting gene lists were compared between HP and AP offspring at the particular stage. The intersection of commonly altered genes between those comparisons represent genes that show the same shift of abundance along stages in both dietary groups and thus likely belong to physiological maturation processes. Therefore, the analysis focused on those transcripts whose change of abundance between two consecutive stages (period I: 94 dpc-1 dpn; period II: 1 dpn-28 dpn; period III: 28 dpn-188 dpn) was limited to either the HP or the AP group. Those represent genes displaying diet-dependent longitudinal transcriptomic alterations.

Between fetal and perinatal stages (period I), 1,750 (605 1 dpn
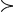
94 dpc in AP) probe sets were altered in level or direction of change of transcript abundance in the AP group compared to the HP group. At 1 dpn, expression of genes associated with RAN signaling and IGF1 signaling was increased; expression of genes participating in organisation of filaments was decreased (Table 2). During the same period, 1,702 probe sets in HP offspring were altered (770 1 dpn
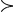
94 dpc in HP) when compared to the AP group. mRNA levels of genes associated with oxidative phosphorylation, purine metabolism, pyrimidine metabolism, and synthesis and degradation of ketone bodies were higher at 1 dpn than at 94 dpc in HP offspring. Conversely, genes participating in glucocorticoid receptor signaling, cyclin and cell cycle regulation, G1/S checkpoint regulation, growth hormone signaling, mitotic roles of Polo-like kinase, and IGF1 signaling were less abundant in HP perinatal piglets.

**Table 2:** Functional annotation of muscle transcripts showing altered abundance between two developmental stages within either dietary group HP or AP (Ingenuity Pathway Analysis).

Developmental comparison	Diet	Affected pathway	Expression	*P* value	No. of genes involved	Genes involved in pathway
94 dpc vs. 1 dpn	AP	RAN signaling	up	8.64*E-3	3	CSE1L, IPO5, KPNA1
	AP	IGF1 signaling	up	3.97*E-2	6	CSNK2A1, MAP2K1, PRKAR2A, RAF1, SOS1, YWHAG
	AP	Organisation of filaments	down	1.26*E-2	13	B4GALT7, BGN, CNP, COL1A2, COL2A1, COL5A1, EVL, FGF2, FNBP1, LOX, MTSS1, TGFB1, TNXB
	HP	Purine metabolism	up	3.19*E-4	21	ADCY3, ATP13A2, ATP6V0B, BCKDHA, BCKDHB, ENTPD4, GMPR, ITPA, MAD2L2, NME1, NME2, NME3, NUDT2, POLD4, POLR1C, POLR2G, POLR2I, POLR2L, PSMC1, RRM2B, RUVBL1
	HP	Pyrimidine metabolism	up	1.19*E-5	16	ENTPD4, ITPA, MAD2L2, NME1, NME2, NME3, NUDT2, POLD4, POLR1C, POLR2G, POLR2I, POLR2L, RPUSD1, RPUSD2, RPUSD4, RRM2B
	HP	Oxidative phosphorylation	up	1.24*E-2	10	ATP6AP1, ATP6V0B, ATP6V0E2, COX6B1, NDUFA8, NDUFB7, NDUFB9, NDUFS3, RPUSD1, TNNI2
	HP	Synthesis and degradation of ketone bodies	up	4.43*E-3	3	ACAA1, ACAT2, BDH1
	HP	Glucocorticoid receptor signaling	down	1.27*E-4	24	CCL2, FOS, GTF2E2, GTF2H3, JUN, NCOA1, NCOA2, NCOA3, NCOR1, PIK3C3, PIK3R3, POLR2D, PPP3CB, PRKACB, RRAS2, SERPINE1, SHC1, SMAD3, STAT1, SUMO1, TBP, TGFB2, TGFBR2, TRAF6
	HP	Cyclin and cell cycle regulation	down	8.09*E-5	12	ATR, CCNA2, CDKN1B, E2F2, HDAC3, HDAC6, PPP2CB, PPP2R1A, PPP2R1B, PPP2R5E, RB1, TGFB2
	HP	G1/S checkpoint regulation	down	1.86*E-3	8	ATR, CDKN1B, E2F2, HDAC3, HDAC6, RB1, SMAD3, TGFB2
	HP	Growth hormone signaling	down	1,71*E-3	9	FOS, GHR, IGF1, IGFBP3, PIK3C3, PIK3R3, RPS6KA3, RPS6KA5, STAT1
	HP	Mitotic roles of Polo-like kinase	down	3.51*E-2	6	CDC27, PLK4, PPP2CB, PPP2R1A, PPP2R1B, PPP2R5E
	HP	IGF1 signaling	down	8.90*E-5	13	FOS, IGF1, IGFBP3, IGFBP5, JUN, PIK3C3, PIK3R3, PRKACB, PTPN11, RRAS2, SHC1, YWHAB, YWHAE
1 dpn vs. 28 dpn	AP	Purine metabolism	up	7.50*E-3	21	ACIN1, ATP11B, ATP13A2, ATP5G2, ATP6V0B, CHRAC1, CILP, DDX19B, DGUOK, NME3, PDE2A, PDE5A, POLG, POLR2D, POLR2E, POLR2F, POLR2J, POLR3K, PRPSAP2, PSMC5, RFC3
	AP	Pyrimidine metabolism	up	5.95*E-3	13	CHRAC1, CTPS2, NME3, POLG, POLR2D, POLR2E, POLR2F, POLR2J, POLR3K, RFC3, RPUSD1, RPUSD4, UCK1
	AP	Organisation of filaments	up	5.74*E-3	12	AKAP2, COL1A1, COL1A2, COL5A2, CRYAA, DBN1, DCN, FN1, FNBP1, PDLIM3D, SERPINH1, TGFB1
	AP	Fatty acid elongation in mitochondria	down	4.39*E-4	5	ACAA2, AUH, HADH, HSD17B4, PECR
	AP	RAN signaling	down	2.49*E-2	3	IPO5, RAN, XPO1
	AP	Oxidative phosphorylation	down	1.54*E-9	25	ATP5B, ATP5C1, ATP5F1, ATP5J, ATP6V1A, ATP6V1B2, ATP6V1C1, ATP6V1H, COX15, COX17, COX6C, COX7C, NDUFA1, NDUFA9, NDUFAB1, NDUFB1, NDUFB3, NDUFB5,NDUFC1, NDUFS2, NDUFV2, PPA2, SDHA, UQCR11, UQCRB
	HP	G2/M DNA damage checkpoint regulation	up	2.63*E-4	8	ATR, CCNB1, CCNB2, PTPMT1, RPRM, WEE1, YWHAE, YWHAG
	HP	Mitotic roles of Polo-like kinase	up	4.95*E-4	9	ANAPC1, ANAPC5, CCNB1, CCNB2, PLK4, PPP2R3A, PTTG1, STAG2, WEE1
	HP	Oxidative phosphorylation	up	2.23*E-3	14	ATP5A1, ATP5O, ATP6V1A, ATP6V1E1, COX7B, FAM63B, IP6K2, NDUFB6, NDUFS1, NDUFS4, NDUFV2, PPA1, PPA2, UHRF1BP1
	HP	RAN signaling	up	2.88*E-3	4	CSE1L, IPO5, TNPO1, XPO1
	HP	Organisation of filaments	down	1.46*E-3	16	ARHGEF2, BGN, COL1A1, COL1A2, COL2A1, COL5A1, DBN1, DCN, EVL, FAT1, FES, LOX, MARK4, NUMA1, PPP1R9AD, SIRPA
28 dpn vs. 188 dpn	AP	AMPK signaling	up	1.10*E-2	11	PIK3R1, PPP2CA, PPP2CB, PPP2R2A, PPP2R5A, PPP2R5E, PRKAA1, PRKAA2, PRKAB2, PRKACB, SMARCA2
	AP	Mitotic roles of Polo-like kinase	up	1.56*E-3	8	ANAPC11, HSP90AA1, PPP2CA, PPP2CB, PPP2R2A, PPP2R5A, PPP2R5E, STAG2
	AP	mTOR signaling	up	9.70*E-4	14	EIF3A, EIF4B, NAPEPLD, PIK3R1, PPP2CA, PPP2CB, PPP2R2A, PPP2R5A, PPP2R5E, PRKAA1, PRKAA2, PRKAB2, RHOQ, RICTOR
	AP	Cyclin and cell cycle regulation	up	6.40*E-4	10	ATR, GSK3B, HDAC2, PPP2CA, PPP2CB, PPP2R2A, PPP2R5A, PPP2R5E, RAF1, TGFB2
	HP	Purine metabolism	up	8.46*E-3	20	AMPD3, ATF7IP, ATP6V0E1, ATP6V1G2, DDX19B, EIF2AK4, MAD2L2, MPP1, NME6, NT5C3, POLG, POLR1A, POLR2C, POLR2F, PPAT, PSMC1, PSMC3, PSMC5, RFC3, VCP
	HP	Pyrimidine metabolism	up	2.36*E-2	11	CMPK1, EIF2AK4, MAD2L2, NME6, NT5C3, NXN, POLG, POLR1A, POLR2C, POLR2F, RFC3
	HP	Glucocorticoid receptor signaling	up	2.13*E-2	16	A2M, AGT, CXCL3, EP300, HSP90AA1, MAP3K14, NCOA2, NCOR2, NR3C1, PIK3C3, POLR2C, POLR2F, SMAD3, STAT5B, TAT, TSC22D3
	HP	Oxidative phosphorylation	down	1.35*E-3	14	COX15, COX6A1, COX7C, IP6K2, NDUFA2, NDUFB3, NDUFB5, NDUFB6, NDUFB10, NDUFS3, NDUFS4, NDUFS6, PPA2, UQCRB
	HP	Mitotic roles of Polo-like kinase	down	2.30*E-2	6	CCNB2, CDK1, PLK4, PRC1, PTTG1, WEE1
	HP	G2/M DNA damage checkpoint regulation	down	2.25*E-2	5	CCNB2, CDK1, CKS1B, WEE1, YWHAZ

Up and down indicate higher and lower abundance in later compared to earlier stages, respectively. P-value: significance of association between dataset and IP-pathways; Fischer's exact test.

When comparing perinatal and infant piglets (period II), expression of 1,864 probe sets was altered in a different manner in AP offspring than in HP offspring. Of these, 900 probe sets were upregulated and 964 probe sets were downregulated by 28 dpn. Genes participating in the organisation of filaments as well as in purine and pyrimidine metabolism were more highly expressed, while genes associated with fatty acid elongation in mitochondria, RAN signaling, and oxidative phosphorylation were decreased during that period in AP offspring. In the same period, 2,130 probe sets exhibited variation that was specific to HP offspring. Of these, 870 probe sets had an increased mRNA level at 28 dpn, including genes involved in G2/M DNA damage checkpoint regulation, mitotic roles of Polo-like kinase, RAN signaling, and oxidative phosphorylation. Decreased mRNA expression was detected for genes associated with the organisation of filaments.

When infant and young adult pigs (period III) were compared, 1,556 probe sets differed significantly (937 188 dpn
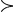
28 dpn in AP) in AP offspring. Increased expression in adult AP offspring was observed for genes participating in AMPK signaling, mitotic roles of Polo-like kinase, mTOR signaling, and cyclin and cell cycle regulation. In HP offspring 1,724 probe sets were differentially expressed (877 188 dpn
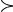
28 dpn in HP). Adult HP offspring had higher expression of transcripts involved in purine metabolism, pyrimidine metabolism, and glucocorticoid receptor signaling. Expression was lower in adult HP offspring for genes involved in oxidative phosphorylation, mitotic roles of Polo-like kinase, and G2/M DNA damage checkpoint regulation.

A comprehensive overview of the pathways observed to be altered between diets (HP vs. AP) and stages (94 dpc; 1, 28, 188 dpn) is depicted in Figure 2.

## Discussion

In a longitudinal study covering prenatal, perinatal, juvenile, and adult developmental stages in a porcine model, we analysed muscle gene expression patterns in offspring from sows fed either an isoenergetic high-protein low carbohydrate or adequate control protein diet throughout pregnancy. Relative mRNA abundances were compared between both dietary groups and developmental stages. Variation of transcript abundances at various pre- and postnatal stages in muscle has been shown [16]–[18]. Accordingly, a longitudinal study was done and evaluated focussing on the interaction of diet x stages in order to consider mechanisms of fetal programming at variable stages-dependent transcriptomic backgrounds. Also impact of IUGR and low birth weight as well as sex on postnatal growth is well documented [19]–[21]; however, our experiment was designed to balance for sex and body weight among the diet-groups at all stages; accordingly, only a few probe-sets were found exhibiting variable abundance due to sex (24 probe-sets) or weight (8 probe-sets). mRNA expression profiles between diets revealed significant shifts at prenatal and postnatal stages. Even more distinct differences were observed between dietary groups when considering temporal changes along development. Thus, the maternal diet produced both short- and long-term transcriptional alterations in the offspring. Because offspring were exposed to standard dietary conditions from birth (through fosters) to adult, in both experimental groups the transcriptional alterations were consequences of the maternal protein supply during prenatal development. In particular, a number of cell cycle modulating pathways and energy producing pathways in muscle tissue were affected by diet.

### Growth and cell maintenance

In mammals cell division is synchronous with cell growth (reviewed in [22], [23]), which is triggered by various factors and functional networks. The fidelity of cell division is monitored in eukaryotic cells by sophisticated mechanisms, including defined checkpoints that initiate cell cycle arrest if processes go awry. Among others, Polo-like kinases (PLK), an evolutionarily conserved family of essential cell cycle regulators, are required at several key points within the cell cycle to ensure entry and exit from mitosis (reviewed in [24], [25]). Cell cycle regulators have been shown to exhibit altered expression at both RNA and protein levels due to varied maternal protein diets [26], [27]. It can be supposed that altered expression of cell cycle regulators will affect cell growth and proliferation. At the tissue and organ level, this could translate into differences in body composition and organ weights. In fact, in two recent studies we reported that excess maternal protein during pregnancy produced slightly smaller pigs with reduced muscularity [13], [14]. Previous work had demonstrated that the number of secondary myofibers present in prenatal skeletal muscle is dependent on the maternal diet [28]. Thus, it appears that maternal diet affects the growth of offspring skeletal muscle prenatally, resulting in some level of IUGR. However, this affect may be transient: we also observed that excess protein supply in sows during pregnancy had only modest effects on the phenotype of juvenile and adult pigs, which only showed slightly lowered muscularity and slight, non-significantly reduced muscle fiber number [13], [14]. Interestingly, juvenile offspring of the HP group had a smaller percentage of STO (slow-twitch oxidative) fibers (P = 0.09) than juvenile AP offspring [14], suggesting a potential impact of the gestational dietary high protein but low carbohydrate intake on primary muscle fiber development. Judged by the metabolic characteristics of the pregnant dams it is possible that HP sows suffer from metabolic energy deficit due to the need for high rates of ureagenesis and gluconeogenesis triggered by the high protein and low carbohydrate intakes [29]. How this is translated to the fetus is difficult to deduce but additionally might suggest specific differences in amino acid supply in the HP offspring (Metzler-Zebeli et al., manuscript under review Br J Nutr [30]). By 188 dpn, though, there were no differences in fiber type frequencies among the dietary groups [14].

Our findings of altered expression in the molecular routes related to cell cycle and organization of filaments are the transcriptional equivalent of the high developmental plasticity exhibited by the offspring, which ensures maintenance of the overall organismal phenotype by adaptive response of cells of the muscle tissue including satellite cells, fat cells, and fibroblasts. Increased gene expression of cell cycle-associated pathways at 94 dpc may indicate possible compensatory growth mechanisms to counteract the observed IUGR.

In HP offspring, the direction of change of cell cycle and growth-associated pathways was stage-dependent. Within developmental period I for HP offspring, transcripts interacting in various cell cycle and growth-related pathways occurred with lower abundance perhaps reflecting an impaired fetal growth performance due to a potential metabolic energy deficit caused by the gestational HP diet [29] and, therefore, contributing to the observed IUGR at birth [2]. In fact, altered expression of genes related to cell cycle and growth regulating pathways might lead to a lengthened cell cycle and culminate in growth retardation [31]. Our findings seem to indicate that, in this regard, a balancing occurs postnatally. In contrast, AP fetuses in period I showed an increased expression of transcripts associated with cell division, including RAN signaling, a positive key regulator of mitosis (reviewed in [32]). During the same period transcripts related to organization of filaments, including transcripts of collagen genes, were more abundant in HP and remained opposed in abundance to that in AP offspring during early postnatal development. Interestingly, the collagen content of porcine skeletal muscle is associated with intrauterine growth, with small littermates having higher collagen content [33], [34].

mRNA expression within developmental period II revealed an increased abundance of transcripts related to cell division-associated pathways in HP offspring, reflecting a kind of compensation that results in the absence of transcriptional differences at 28 dpn. These transcriptional changes parallel development at the organismal level, with newborns of the HP group having a significantly lower birth weight and lower body fat content than newborns of the control group. However, neither body weight or body composition nor cellularity of muscle and adipose tissue of weaning piglets at 28 dpn showed any differences [13].

### Transcriptional alterations regarding energy metabolism and lipid metabolism

Recent studies have demonstrated that metabolic health is highly dependent on mitochondrial integrity, including proper energy production [35]–[37]. For developmental and metabolic processes, effective generation of ATP is required, which occurs most efficiently via oxidative phosphorylation (OXPHOS) in mitochondria [38]. In rodents and pigs mitochondria are sensitive, at the transcriptional level, to prenatal dietary modifications [9], [39], [40] and the maternal metabolic status [41]. Here, we consistently observed that a maternal HP diet led to increased mRNA expression levels of OXPHOS-related genes in muscle tissue, which may suggest a transiently lower energy status or higher energy demands leading to an adaptive upregulation of processes leading to ATP synthesis. Interestingly, the expression pattern of genes related to OXPHOS was biphasic across temporal development in HP offspring. Increased levels of OXPHOS-associated genes within developmental periods I and II may indicate increased metabolic activity as a postnatal short-term response in muscle tissue of HP offspring as a result of an exposure to energy deficient intrauterine environment. Therefore, after the prenatal ‘steady state’ in terms of metabolic activity induced by the maternal HP diet, compensatory mechanisms may occur at early postnatal stages in HP offspring. This phenomenon reflects a high developmental plasticity of HP offspring. However, at adulthood decreased expression levels of genes related to OXPHOS may account for alterations in mitochondrial activity in HP offspring. This is consistent with findings in IUGR rats, which showed an impaired oxidative phosphorylation as adults [42]. Interestingly, within developmental period III a higher abundance of transcripts related to AMPK (AMP-dependent activated protein kinase) signaling was observed in AP offspring. Because AMPK is a potent activator of mitochondrial metabolism [43], the observed expression pattern may account for metabolic health in AP offspring but not in HP offspring at the adult stage. Taken together, these findings suggest impairments in energy metabolism in muscle tissue of HP offspring along development.

Skeletal muscle is the main peripheral tissue functioning in fatty acid oxidation. However, at the transcript level only a few metabolic pathways related to lipid metabolism were altered in muscle tissue in response to diet. This finding supports the assumption that lipid metabolism-related genes are targets of fetal programming in liver tissue [9] rather than in peripheral tissues like skeletal muscle.

### Transcriptional alterations regarding stress response

Biological effects of glucocorticoids, including metabolic, behavioural, cardiovascular, and immune functions, are transmitted via glucocorticoid receptors and related downstream signaling molecules. In rodents maternal diet affects mRNA expression of the glucocorticoid receptor and its signaling molecules [44]–[46]. Consistently, a diet-dependent alteration in glucocorticoid receptor signaling was also found in porcine offspring [9]. Our similar finding of higher abundance of transcripts related to glucocorticoid receptor signaling within developmental period III may indicate that adult HP animals were in a kind of alarm state with possible side effects on health and metabolism.

### Conclusions

The analysis of longitudinal changes of transcript abundance in skeletal muscle in response to gestational diets with differing protein∶carbohydrate ratios revealed both short- and long-term effects. According to expression profiles of HP offspring, alterations of the transcriptome relevant to pathways of growth and cell cycle regulation were modified in response to diet. These alterations might be related to both IUGR and postnatal compensatory effects. Given that dietary effects on the organismal phenotype in terms of body weight, body composition, and cellularity of muscle and adipose tissue were very modest and only transient, the observed transcriptional alterations represent adaptive processes. In liver modulation of energy producing and sensing pathways was observed [9]; in skeletal muscle, however, shifts in molecular routes related to cell cycle are predominant throughout development. Also the temporal sequences of shifts differ between the central metabolic organ, the liver, and the peripheral tissue of muscle. In muscle, differences of the transcriptomes of HP and AP offspring are highest at 94 dpc and 1 dpn, i.e., time points that are important to myogenesis. At later stages the differences observed in longitudinal shifts lead to a minimization of differences between AP and HP offspring phenotypes at 28 and 188 dpn. In contrast, in liver at a late prenatal stage a steady state was observed, and at later stages a significant modulation and different responsiveness of the transcriptome was observed [9]. Overall the study indicates that pigs show a high level of developmental plasticity that allow adaptation to a high protein∶low carbohydrate maternal environment possibly related to metabolic energy deficiency *in utero* on the organismal level, paralleled by an enduring modulation of the transcriptome. However, the altered mRNA abundance of genes related to energy metabolism may indicate a higher susceptibility of offspring to metabolic challenges, leading to predisposition for metabolic disturbances at later adulthood when offspring is exposed to physiological or nutritional challenging situations.

## Materials and Methods

### Animals and sample collection

**Figure 3:**
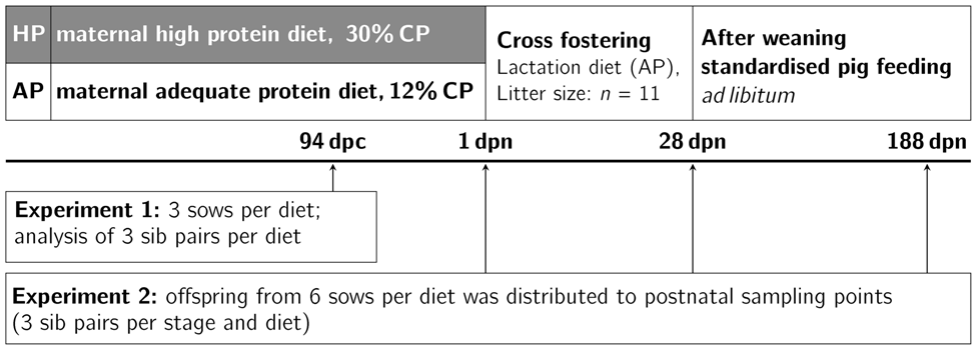
Experimental design. Fetuses and offspring of divergently fed sows were collected at 4 developmental stages. Fetuses were derived from 3 sows per dietary group. Offspring were full sibs of six litters per dietary group collected at 3 consecutive postnatal stages; HP = high protein, CP = crude protein, AP = adequate protein.

Animal care and tissue collection were performed according to guidelines of the German Law of Animal Protection and with approval by the Animal Care Committee of the State Mecklenburg-Vorpommern (Landesamt fr Landwirtschaft, Lebensmittelsicherheit und Fischerei, Mecklenburg-Vorpommern, Germany; LVL MV/TSD/7221.3-1.1-006/04; LALLF M-V/TSD/7221.3-1.2-05/06; LALLF M-V/TSD/7221.3-1.2-013/06). Experimental diets were administered as described [2]. Briefly, at insemination German Landrace primiparous sows (n = 42) were randomly assigned to either a high-protein diet (HP) with 30% (w/w) crude protein or an adequate-protein diet (AP) containing 12% crude protein. Diets were formulated to be isoenergetic (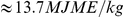) by adjustment of the carbohydrate component of the diet (HP; 30% crude protein; protein: carbohydrate ratio 1∶1.3; AP; 12% crude protein; protein: carbohydrate ratio 1∶5) [2]. Tissue sampling included offspring of these sows at one prenatal [94 days post conception (dpc)] and three postnatal [1,28, 188 days post natum (dpn)] time points (Figure 3).

At 94 dpc, a subset of three sows per dietary group was subjected to Caesarean section. Eight viable fetuses per sow were collected starting at the tip of the left uterine horn and alternating between left and right horn. Fetuses were killed by i.v. injection of T61 in the *V.cava cranialis* and muscle samples (*Musculus longissimus dorsi*) were immediately collected (approximately 500 mg), frozen in liquid nitrogen, and stored at 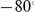
C until analysis. All litters sampled comprised at least 11 viable piglets. There were no differences in the number of fetuses per litter between the gestation diet groups. Fetuses of HP fed dams had a lower mean weight compared to AP fetuses at 94 dpc (HP: 608
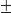
107 g, and AP: 701
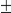
103 g, respectively; P = 0.16; n = 12). The lightest and the heaviest fetuses within one litter were selected for transcriptome analysis.

Offspring collected for postnatal time points were born to primiparous sows after prostaglandin induction of parturition as described [2] and were farrowed after mean pregnancy duration of 115 days. Offspring of six sows per diet with a minimum of 11 liveborn piglets (median litter size = 13) were used; litter size was not different among groups. At birth 10 piglets in each litter were distributed to groups for three time points (1, 28, and 188 dpn). For microarray analyses, six sex-balanced sib pairs (all stages) were chosen per stage and diet. At 1 dpn the lightest and the heaviest piglets within one litter were selected; at later stages body weight was not a criterion for selection but was recorded for consideration in the statistical evaluation. Considering all piglets derived from the complete experiment, mean birth weights of HP newborn piglets were lower than birth weights of AP offspring (HP: 1.21
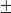
0.04 kg, and AP: 1.41
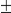
0.04 kg respectively, P
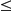
0.05) [2], [13]. However, in the smaller subset of samples used for microarray analyses, differences in body weights between HP and AP offspring did not reach statistical significance (HP: 1.27
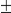
0.32 kg, and AP: 1.28
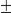
0.43 kg, respectively; P = 0.96; n = 12). Thirty-six hours after birth, the lightest and the heaviest piglet within one litter were killed by i.m. injection of 1.25 mg propionyl-promazine (0.2 ml Combelen, Bayer AG, Leverkusen, Germany) and 50 mg ketamine (Ursotamin, Serumwerk Bernburg AG, Germany). Samples were immediately collected from *M. longissimus dorsi* (approximately 500 mg), frozen in liquid nitrogen, and stored at 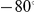
C until analysis.

Remaining piglets were cross-fostered to multiparous sows, which were fed AP diets during gestation and lactation. Litter size during suckling was standardized to 11 piglets per sow. Male piglets were castrated at 4 dpn. From weaning (28 dpn) to slaughter (188 dpn), all piglets were individually reared. They had free access to standard diets formulated for postweaning (29 to 76 dpn), growing (77 to 105 dpn), and finishing periods [13], [14]. At 28 dpn (HP: 7.98
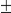
1.51 kg, and AP: 7.78±
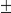
2.31 kg respectively; P = 0.86; n = 12) and 188 dpn of age (HP: 130.57
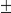
8.01 kg, and AP: 132.47
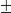
19.58 kg respectively; P = 0.83; n = 12), pigs were weighed after an overnight fast and killed by electronarcosis followed by exsanguination in the experimental slaughterhouse of FBN. Muscle tissue was immediately collected from M. longissimus dorsi, frozen in liquid nitrogen, and stored at 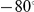
C until use for RNA isolation.

### RNA isolation, target preparation, and hybridization

Total RNA from individual muscle samples was isolated using Tri-Reagent (Sigma-Aldrich, Taufkirchen, Germany) and subsequently subjected to DNase treatment and a column-based purification using the RNeasy Mini Kit (Qiagen, Hilden, Germany). RNA integrity and quantity were checked by agarose gel electrophoresis and by spectrometry with a NanoDrop ND-1000 spectrophotometer (PEQLAB, Erlangen, Germany). Absence of a DNA contamination was verified by PCR of the porcine glyceraldehyde-3-phosphate dehydrogenase (GAPDH) gene (Forward primer: AAGCAGGGATGATGTTCTGG; Reverse primer: ATGCCTCCTGTACCACCAAC) with isolated RNA as template. All RNA samples were stored at 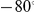
C until downstream analysis. For the microarray experiments individual biotin-labeled cDNA was synthesized by the Gene Chip 3′ Express Kit (Affymetrix, Santa Clara, CA, USA). cDNA was fragmented (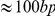) and hybridized on Affymetrix GeneChip Porcine Genome Arrays. After staining and washing steps the arrays were scanned (Affymetrix, Santa Clara, CA, USA).

### Data analysis

Bioinformatic analysis was done in R [47]. First, a quality control was performed. Except for the AP group at 188 dpn (4 samples), all diet group and stage combinations had 6 samples meet the appropriate quality control criteria. Samples were GC-RMA normalized (Log2) over all stages. The MAS5 algorithm was used to skip those transcripts expressed in less than 50% of the animals within one dietary group per stage. For a second filtering step standard deviations were calculated for each probe set over all subsets of arrays of the particular comparisons. Probe sets with a low standard deviation (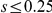) were discarded because such transcripts are not likely to show an altered abundance. Relative changes in mRNA levels were determined using a mixed model analysis, including effects of dietary treatment, stage, sex, weight (as deviation from the mean weight within stage, in percent), and interaction between diet and stage, as well as mother as a random effect. P-values (significance set at 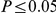) for each comparison were converted to a set of q-values (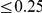) using the algorithm proposed by Storey and Tibshirani [48].

Throughout the manuscript, results are given for the comparisons in the direction of HP vs. AP; thus ‘increased abundance’ indicates higher transcript abundance in HP than in AP. Analysis of the pathways involved was carried out using Ingenuity Pathway Analysis [49]. The up-to-date annotation of Affymetrix probe sets to EnsEMBL Sscofa 9 (20,439 of 23,935 annotated probe sets) was used [15]. All the microarray data are MIAME compliant, and the raw data have been deposited in a MIAME-compliant database, the National Center for Biotechnology Information Gene Expression Omnibus (www.ncbi.nlm.nih.gov/geo) (accession numbers: GSE33737, GSE33738).

### Pathway analysis

Gene lists from microarray results were submitted to Ingenuity Pathways Analysis (Ingenuity) to assign the altered genes to biofunctions and canonical pathways. The focus was on those canonical pathways appearing at least once within the top ten altered pathways within one single analysis. Interactions presented in the networks are not specific for porcine muscle tissue, as the database contains literature from many different research areas.
